# Candidate l‐methionine target piRNA regulatory networks analysis response to cocaine‐conditioned place preference in mice

**DOI:** 10.1002/brb3.2272

**Published:** 2021-07-01

**Authors:** Kunlin Zhang, Guanyu Ji, Mei Zhao, Yan Wang

**Affiliations:** ^1^ Institute of Psychology CAS Key Lab of Mental Health Beijing China; ^2^ Department of Psychology University of Chinese Academy of Sciences Beijing China; ^3^ ShenZhen Gendo Health Technology Co., Ltd ShenZhen China

**Keywords:** cocaine, CPP, gene expression, high‐throughput sequencing, piRNA

## Abstract

**Background:**

Methionine has been proven to inhibit addictive behaviors of cocaine dependence. However, the mechanism of methionine response to cocaine CPP is unknown. Recent evidence highlights piRNAs to regulate genes via a miRNA‐like mechanism. Here, next‐generation sequencing is used to study mechanism on methionine response to drug‐induced behaviors though piRNA.

**Methods:**

l‐methionine treatment cocaine CPP animal model was used to do non‐coding RNA sequencing. There were four groups to sequence: saline+saline (SS), MET+saline (MS), MET+cocaine (MC), and cocaine+saline. Combining mRNA sequencing data, the network and regulation of piRNA were analyzed with their corresponding mRNA and miRNA.

**Results:**

Analysis of the piRNAome reveals that piRNAs inversely regulated their target mRNA genes. KEGG analysis of DE‐piRNA target mRNA genes were enriched in Morphine addiction, GABAergic synapse and Cholinergic synapse pathway. Furthermore, four significantly differential expressed genes Cacna2d3, Epha6, Nedd4l, and Vav2 were identified and regulated by piRNAs in the process of l‐methionine inhibits cocaine CPP. Thereinto, Vav2 was regulated by multiple DE piRNAs by sharing the common sequence: GTCTCTCCAGCCACCTT. Meanwhile, it was found that piRNA positively regulates miRNA and three genes Bcl3, Il20ra, and Insrr were identified and regulated by piRNA through miRNA.

**Conclusion:**

The results showed that piRNA negatively regulated target mRNA genes and positively regulated target miRNA genes. Genes located in substance dependence, signal transduction and also nervous functions pathways were identified. When taken together, these data may explain the roles of l‐methionine in counteracting the effects of cocaine CPP via piRNAs.

## Graphical abstract

our results showed piRNA negatively regulated target mRNA genes and positively regulated target miRNA genes. Genes located in substance dependence, Signal transduction and also nervous functions pathways were down‐regulated: Cacna2d3, Epha6, Nedd4l and Vav2. Thereinto, Vav2 was targeted by multiple piRNAs by sharing the common target sequence: GTCTCTCCAGCCACCTT. Taken together, these data may explain the roles of L‐methionine in counteracting the effects of cocaine CPP via piRNAs.

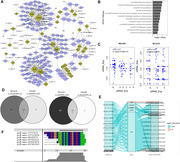

## 1 INTRODUCTION

Addictions are a diverse set of common, complex diseases that are to some extent tied together by shared genetic and environmental etiological factors. Cocaine affects many neurochemical systems. Previous studies have further demonstrated that PFC‐dependent processes, such as executive function, explicit learning, and memory, are damaged in animal models of cocaine addiction and in human cocaine abusers (Chao et al., 2014). Knowledge of genetic factors in etiology and treatment response to cocaine addiction may enable the individualization of prevention and treatment, as well as the identification of new therapeutic targets.

Non‐coding RNAs (ncRNAs) have been gaining recognition for their involvement in genetic and epigenetic regulation (Cech & Steitz, 2014). A large body of studies have reported the distribution of piRNAs in brains, including the hippocampus. Increasing evidence suggests that piRNAs play a critical role in both epigenetic and post‐transcriptional silencing of transposons (Siomi et al., 2011; Thomson & Lin, 2009). piRNAs are shown to direct DNA methylation on a non‐transposon locus to regulate genomic imprinting and influence synaptic plasticity of neuronal cells through DNA methylation on non‐transposon genomic regions (Rajasethupathy et al., 2012; Watanabe et al., 2011). There is evidence that the Piwi/piRNA complex in the *Aplysia* brain was sensitive to serotonin modulation, regulates CREB2 and genes to control nervous system function via a miRNA‐like mechanism that operates by imperfect base‐pairing rules (Aravin et al., 2001; Rouget et al., 2010; L. Zuo et al., 2016). Hence, conducting piRNAome analysis is very important for uncovering mechanism of drug abuse.

Epigenetic changes, specifically alterations in the pattern of DNA methylation, which can produce long lasting alterations in gene expression affecting behavior, have been verified by mounting reports (Maze & Nestler, 2011; Tsankova et al., 2007). l‐methionine (MET) as a dietary methyl donor is the major driver of DNA methylation (N. Zhang, 2018). MET has partitioned functions among protein synthesis, redox balance, polyamine biosynthesis, and the de novo pathway (also referred to as the methylation cycle or recycling pathway) where it is converted to S‐adenosyl methionine (SAM), a principal methyl donor (Bolander‐Gouaille & Bottiglieri, 2007; Sanderson et al., 2019). MET is involved in the synthesis of various neurotransmitters in the brain. It is considered to be the principal sulfur‐containing amino acid in proteins, and play critical roles in nurture‐based metabolism with brain development and functioning (Ringman & Coppola, 2013; Vuaden et al., 2012). Numerous evidence indicates that dietary methionine restriction is associated with increased longevity and decreased incidence of age‐related disorders in mice and rats (Malloy et al., 2006; Miller et al., 2005; Zimmerman et al., 2003). More importantly, our initial findings and that of others suggest that methionine administration can inhibit addictive behaviors in rodent models of cocaine dependence (Tian et al., 2012, 2016; Wright et al., 2015). Currently, the specific mechanisms of methionine effects on drug‐induced behaviors remain unclear.

Based on this evidence, the goal of this study was to identify the mechanisms of MET inhibitory effects on cocaine‐induced cellular and behavioral changes through piRNAs. In the current study, it is the first time we identified Vav2 genes regulated by multiple piRNAs in the PFC upon cocaine treatment and reversed by l‐methionine. The novel targets will be useful for further therapeutic intervention.

## 2 MATERIALS AND METHODS

### 2.1 Animals housed, drug treatment and CPP test

Animals housed, drug treatment and CPP were as described before (Wright et al., 2015). Briefly, adult male C57/BL6 mice (20–30 g) were housed under a 12 h light/dark cycle. Mice were injected subcutaneously with 1 g/kg (6.6 mmol/kg) l‐methionine twice a day for 10 consecutive days using the CPP procedure. During training, mice were injected with methionine 1 h before each behavioral experiment.

Mice were paired up for 8 days with the saline group receiving saline in both sides of the chambers, and drug groups were injected with cocaine (20 mg/kg, i.p., Qinghai pharmaceutical group co. LTD, china) and saline on one side and drug only on the opposite side (Romieu et al., 2008). After each manipulation, the mice were confined to the corresponding conditioning chambers for 30 min before being returned to their cages. On test day, place preference score (CPP score) was assigned by subtracting the time spent in the drug paired chamber from the time spent in the saline paired chamber. Animals were divided into 4 groups: (1) saline+saline (SS), (2) MET+saline (MS), (3) MET+cocaine (MC), and (4) cocaine+saline (CS).

### 2.2 RNA extraction

Two hours after the (day 10) CPP test, the animals were killed (being anesthetized with dry ice) and their mPFCs surgically excised. The tissue was stored in liquid nitrogen immediately after extraction and then transferred to the −80°C freezer. Total RNA was extracted from the frozen tissues using the TIANamp DNA/RNA isolation kit (TIANGEN), including additional treatment with RNase‐free DNase I (Ambion) for 30 minutes at 37°C to remove contaminating DNA.

### 2.3 piRNA sequencing data

In this study, piRNA sequencing data was separated from ncRNA sequencing data. 1 μg of total RNA from a pool of 2 animals (500 ng each) was used to separate and recover 18–30 nt RNA segments by PAGE gel. Then the standard step was followed for generating sequencing library from the addition of poly‐A. Sequencing was performed with single‐end reads of 50 bp through the BGISEQ‐500 platform at Beijing Genomics Institute (BGI; Shenzhen, China). Three biological replicates were sequenced for each group.

### 2.4 piRNA bioinformatics

Raw sequencing reads were filtered to remove low quality, adapter contaminated, shorter than 16 nt reads with FASTQ (Cock et al., 2010). Clean reads were mapped to the reference genome by using AASRA (Tang et al., 2017). The results matching to miRNA, rRNAs and tRNAs were excluded. The remaining reads were aligned against piRBase (J. Wang et al., 2019) using Bowtie allowing one mismatch. In order to investigate the expression profiles of piRNAs, the frequency of piRNA counts were normalized to TPM (tags per million) using the following formula: normalized expression = actual miRNA read count /total clean read count × 10^6^.

Differentially expressed piRNAs between the paired groups were analyzed by using DEGseq (L. Wang et al., 2010). The *p*‐values calculated for each gene were adjusted to *Q*‐values for multiple testing corrections by two alternative strategies (Benjamini & Hochberg, 1995; Storey & Tibshirani, 2003). To improve the overall accuracy of DEGs results, a gene was defined as a DE‐piRNA (differentially piRNA) when *Q*‐values≤ .05.

### 2.5 piRNA target prediction

Recent evidence has suggested interaction between piRNAs and mRNAs through base‐pair complementarity and a possible inverse correlation between piRNA expression and its corresponding mRNA targets (Mao et al., 2019; Y. Zuo et al., 2019). In this study, we get piRNA target prediction of mRNA and miRNA from piRBase (J. Wang et al., 2019) with rules described as before: base pairing at the 5′ end of piRNA with mismatches of <3 in the following 20‐nt sequence (P. Zhang et al., 2015).

### 2.6 Real‐time PCR validation

Validation of DEGs was performed by quantitative reverse‐transcription PCR (RT‐qPCR) using the Maxima SYBR Green qPCR Master Mix kit (Fermentas), according to the manufacturer's instructions, in an ABI Prism 7500 Sequence Detection System machine (Applied Biosystems Inc.). All real‐time RT‐qPCR data were normalized to SS expression using forward and reverse primers listed in Supporting Information Table S1.

### 2.7 Statistical analysis

Data are expressed as the mean ± SD. Statistical analysis of behavior data and qPCR was performed using an unpaired t test with two tailed distributions. The results were considered statistically significant when *p* < 0 .05.

Cytoscape (3.8.0) was used to compute enriched gene networks. Heatmaps were created using Morpheus (https://software.broadinstitute.org/morpheus/).

### 2.8 Ethics approval

This study has been approved by the ethics committee of the Institute of Psychology, CAS.

## 3 RESULTS

### 3.1 Effects of methionine on cocaine‐induced behavior

**FIGURE 1:**
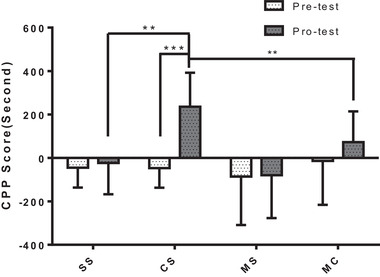
Effect of l‐methionine (MET; 200 mg/kg) on behavior in response to cocaine‐addicted mice (20 mg/kg, i.p.). Abbreviations: CS, cocaine+saline; MC, MET+cocaine; MS, MET+saline; SS, saline+saline. Note: All results represent the mean ± SD of 10 independent determinants (*n* = 10/group) in behavioral test experiments. ***p < 0.001, **p < 0.01, *p < 0.05

As shown in Figure 1, there were significant differences in cocaine‐induced CPP among groups of saline and cocaine CPP with or without methionine treatment (*p* < 0.001). Meanwhile, methionine administration significantly attenuated cocaine‐CPP behavior (CS vs. MC *p* < 0.01).

### 3.2 Characterization of ncRNA sequencing

**FIGURE 2:**
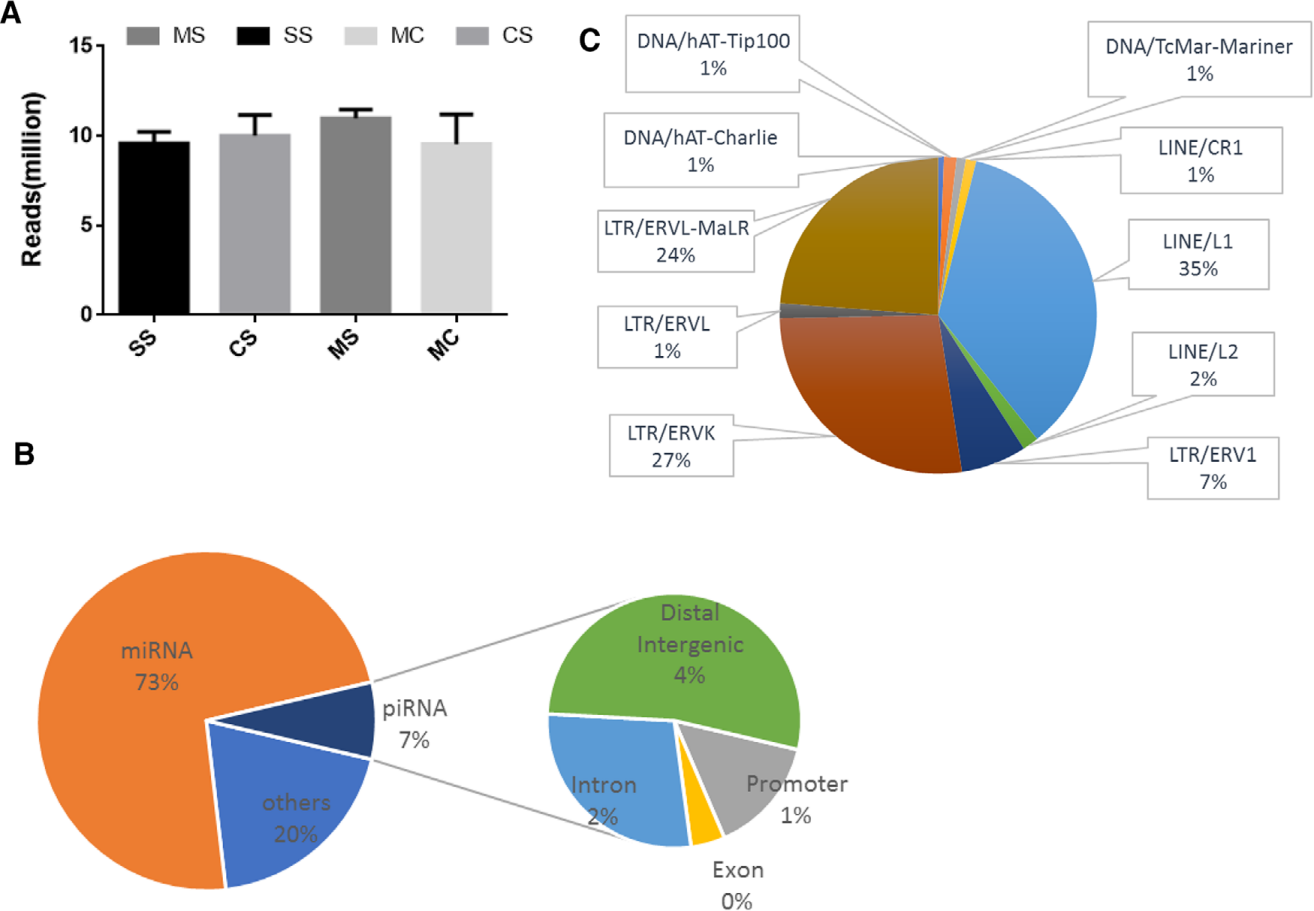
Characterization of small RNA sequencing. (a) Average sequencing reading of each sequencing sample. (b) Non‐coding RNA distribution of the sequencing data and distribution of annotation elements of piRNAs. (c) Distribution of annotation elements of repetitive elements of piRNAs

To understand the molecular mechanisms underlying the effects of cocaine CPP and delineate the functional consequences of methionine treatment, we performed ncRNA sequencing of the extracted PFCs of mice from each of the four treatment groups: SS, CS, MS, and MC. We obtained on an average 10 million clean reads for each sample. There was no significant difference of the reads among each group of the total reads (Figure 2a). Annotation showed that 73% were miRNA, 7.3% were piRNA and the left were other snoRNA, genomic, tRNA and et al., indicating that small RNA content was dominated by miRNAs and piRNAs. Of these sequenced piRNAs, most were located in repeated regions, intron and a small part in promoter and exon (Figure 2b). While in the repeated regions, most were located in LINE1 (35%), LTR/ERVK (27%), and LTR/ERVL‐MaLR (24%, Figure 2c). The result was coinciding with the report that piRNAs clusters are often located in intergenic regions (L. Zuo et al., 2016).

### 3.3 The response of MET on cocaine CPP

**FIGURE 3:**
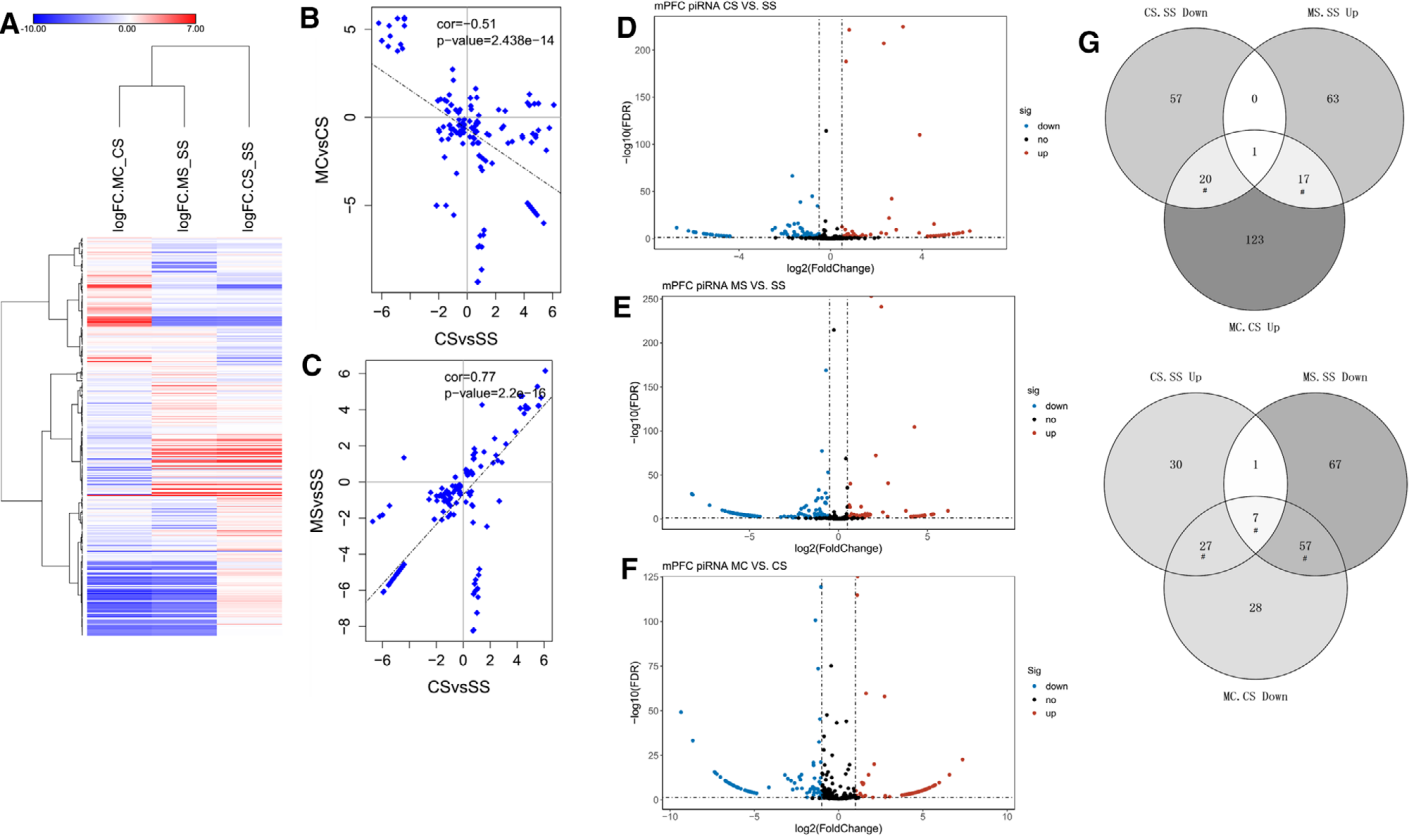
l‐methionine reverses broad changes of piRNA expression caused by cocaine. (a) Heat map of all significantly different piRNA genes in each group (saline+cocaine [CS], MET+saline [MS], and MET+cocaine [MC]). Red, up‐regulated; blue, down‐regulated. Scatter plot of relationship of regulation of piRNAs between CS and MC groups (b), between CS and MS groups (c). (d–f) Volcano plot of differentially regulated piRNAs of cocaine+saline (CS), methinione+saline (MS), and methinione+cocaine (MC) (*p* < .05). Up‐regulated DE_piRNAs are represented by red and down‐regulated by blue. The log2 fold change values for CS vs. SS, MS vs. SS and MC vs. CS are plotted against the average log expression values, respectively. (g) Venn diagrams of the significantly up‐regulated and down‐regulated piRNAs among three different treatment groups (CS, MS, and MC)

Behavior test above showed that MET attenuated behaviorally cocaine‐CPP which induced the question whether MET clearly reversed cocaine‐induced piRNA genes. We performed ncRNA sequence and first plotted the log_2_fold_change of piRNAs from CS, MS, and MC conditions in a heatmap. As expected, a significant number of genes exhibited fold changes in opposite directions in the MC mice compared with CS (Figure 3a,b, *p*‐value: 2.44e‐14). This indicated that l‐methionine reversed piRNAs induced by cocaine. When we compared piRNAs of CS and MS groups, there is a positive relationship between them (Figure 3a,c). Considering that l‐methionine did not induce any abnormal behavior, piRNAs induced by l‐methionine treatment cannot be considered to relate to cocaine response.

### 3.4 Broad effect of cocaine and l‐methionine on piRNAome

To investigate the effect of piRNA of saline and MET response to drug, differentially expressed piRNAs analysis of the ncRNA sequencing data was performed. There were 143 differentially expressed piRNAs (DE_piRNAs) in the CS group compared to the SS group (Figure 3d). Of them, 65 genes were up‐regulated and 78 genes were down‐regulated following cocaine CPP. When compared with the SS group, the MS group showed 67 piRNAs significantly high regulated, and 132 piRNAs were significantly low regulated (Figure 3e, Supporting Information Table S2).

In order to study the effects of combined cocaine and MET treatment on piRNAs, we applied the same analyses to find piRNAs of which the expression was altered by repeated cocaine treatment, with the condition that differential expression is inhibited by MET treatment. We performed a differential expression analysis between MC and CS groups to obtain a list of DE‐piRNAs affected by MET treatment in the context of cocaine treatment. A total of 139 DE‐piRNAs were found up‐regulated and 119 DE‐piRNAs down‐regulated in the MC‐treated mice when compared with that of CS‐treated mice (Figure 3f).

Then, we examined how behavioral differences between the effects of cocaine, MET and cocaine combining MET might be explained partially by the differences of piRNAs. The Venn diagram in Figure 3g reveals that there is significant overlapping of DE‐piRNAs between CS_SS and MC_CS (*p* < 1.468e‐07; *p* < 8.852e‐31) and between MS_SS and MC_CS (*p* < 1.987e‐05; *p* < 5.435e‐54) but not between CS_SS and MS_SS, which reinforce the speculation that l‐methionine treatment was not related to cocaine response in Figure 2c. Interestingly, there is a significant overlap of DE‐piRNAs among up‐regulated CS_SS, down‐regulated MS_SS and down‐regulated MC_CS (*p* < 6.707e‐05) indicating that behavioral changes as a result probably affected cocaine‐induced certain piRNAs which reverses by MET.

### 3.5 piRNAs target mRNA gene prediction

**FIGURE 4:**
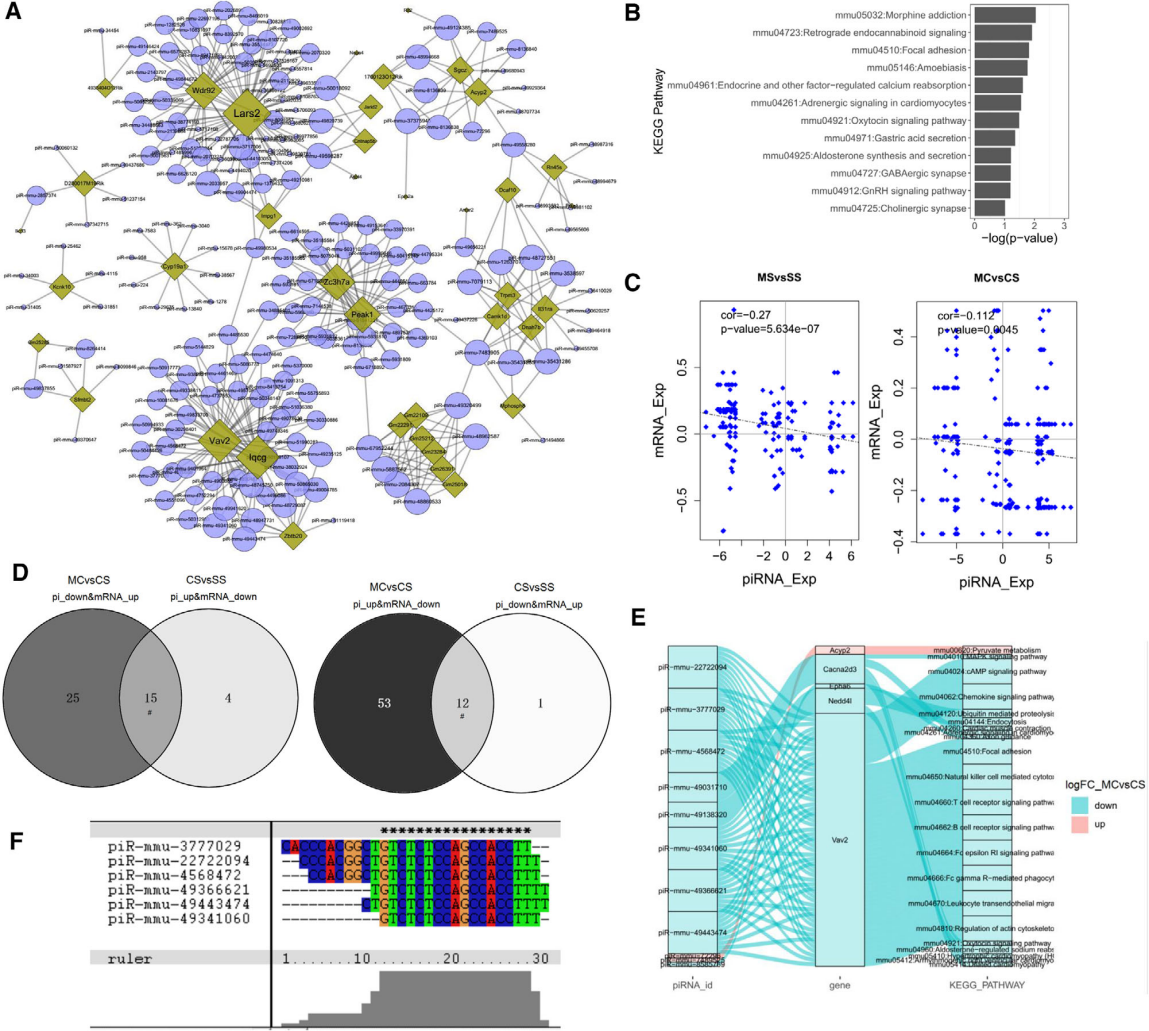
Interactions between piRNA and mRNA of cocaine addiction and MET treatment response to cocaine addiction. (a) Gene regulatory network of significantly regulated piRNAs and their potential target mRNA genes. (b) Chart of enriched pathways of target mRNA genes of De‐piRNAs. (c) Scatter plot of relationship of regulation between piRNAs and their potential target mRNA genes of MSvsSS group and MCvsCS group. (d) common piRNAs with reversely regulated DEGs between MCvsCS and CSvsSS. (e) Sankey diagram of DE‐piRNA acted by MET treatment response to cocaine addiction with their target DEGs and pathways. (f) Common sequence of multiple piRNA sequence alignments of the predicted target Vav2 gene

Recent evidence suggests that piRNAs, in a mechanism similar to miRNAs, may regulate gene expression through base pair complementarity with their targets (Chu et al., 2015; Hashim et al., 2014). However, few studies have identified the corresponding gene targets of specific piRNAs on drug abuse. According to base pair complementarity rules, to evaluate gene expression from a network perspective and gain further insight into the mechanism by which piRNA changes might influence gene expression, we used our mRNA sequencing data (GEO: GSE146631) and performed network by significant piRNAs and their potential target genes with cytoscape (v3.8.2) (Figure 4a). There are several target genes enriched by piRNAs such as Wdr92, Lars2, Iqcg, Vav2 and so on. Then we used those target mRNA genes to do pathway analysis with DAVID (da Huang et al., 2009), it showed that some pathways enriched in cases of substance dependence (morphine addiction) and nervous functions pathways (GABAergic synapse and cholonergic synapse) which are reported to be closely associated with drug addiction (Figure 4b).

To unclose the relationship between piRNAs and their potential target mRNAs, the plot of log_2_fold_change of piRNA and the potential target genes was conducted and showed that there is a significant negative correlation between piRNA and the potential target mRNA genes of MSvsSS group (cor: −0.27, *p*‐value: 5.63E‐07) and MCvsCS group (cor: −0.11, *p*‐value: 0.0045) (Figure 4c). This was in coincidence with the published possible mechanism regulation of piRNAs to their target mRNA genes (Hashim et al., 2014; Krishnan et al., 2017).

### 3.6 MET acts on piRNAs to down‐regulate genes located in addiction‐related pathways induced by cocaine

Opposite directions of fold changes of genes in the MC mice compared with CS shown in Figure 2b and negative relationship of log_2_fold_change between piRNA and the potential target genes shown in Figure 4c indicated that methionine contacted the effect of cocaine by acting on piRNAs to regulate mRNAs. To further investigate target genes of MET treatment response of cocaine CPP regulated by piRNA, we did overlap piRNAs changed in opposite direction with targeting genes between MC and CS groups (Figure 4d). There is a highly significant (*p* < 6.18e‐09) overlap of piRNAs up‐regulated by cocaine and down‐regulated by MET with target genes in opposite direction, and also highly significant (*p* < 6.34e‐06) overlap piRNAs down‐regulated by cocaine and up‐regulated by MET with target genes in opposite direction. Then we did pathway analysis with these target genes and plotted in Sankey diagram (Figure 4e). It showed that except Acyp2 gene, Cacna2d3, Epha6, Nedd4l and Vav2 were down‐regulated by MET via piRNA when induced by cocaine. And as for pathways these genes located such as mmu04024: cAMP signaling pathway, mmu04010: MAPK signaling pathway, mmu04360: Axon guidance and so on were reported related to drug dependence (Hope et al., 2007; Y. Wang et al., 2018).

It was noted that there were multiple piRNAs induced by MET down‐regulated Vav2. Vav2 is an important gene located in multiple pathways report associated with drug addiction such as mmu04024: cAMP signaling pathway, mmu04062: Chemokine signaling pathway (Cui et al., 2014), mmu04510: Focal adhesion (Verma et al., 2019) and so on as a hub. Identifying recurring sequence patterns is very important in order to understand how piRNA binds and controls its target mRNA. We used clustalX (http://www.clustal.org/) to do sequence alignment of the piRNAs and showed the common sequence: GTCTCTCCAGCCACCTT as the target sequence works on (Figure 4f). Taken together, these evidences suggest that cocaine exposure first inhibits piRNAs and then regulated Vav2 genes via common target sequence and MET reverses piRNAs and their target genes.

### 3.7 piRNAs target miRNA prediction

It is reported that miRNA can regulate the expression of protein‐coding mRNA transcripts by binding to the 3′ untranslated region (3′ UTR) of target transcripts and blocking their translation (Kenny, 2014). There are few papers which studied a combined miRNA–piRNA regulation or network to detect diseases (Jain et al., 2019). In current paper, we tried to draw the regulatory network of how piRNA regulates protein‐coding genes via miRNAs.

**FIGURE 5:**
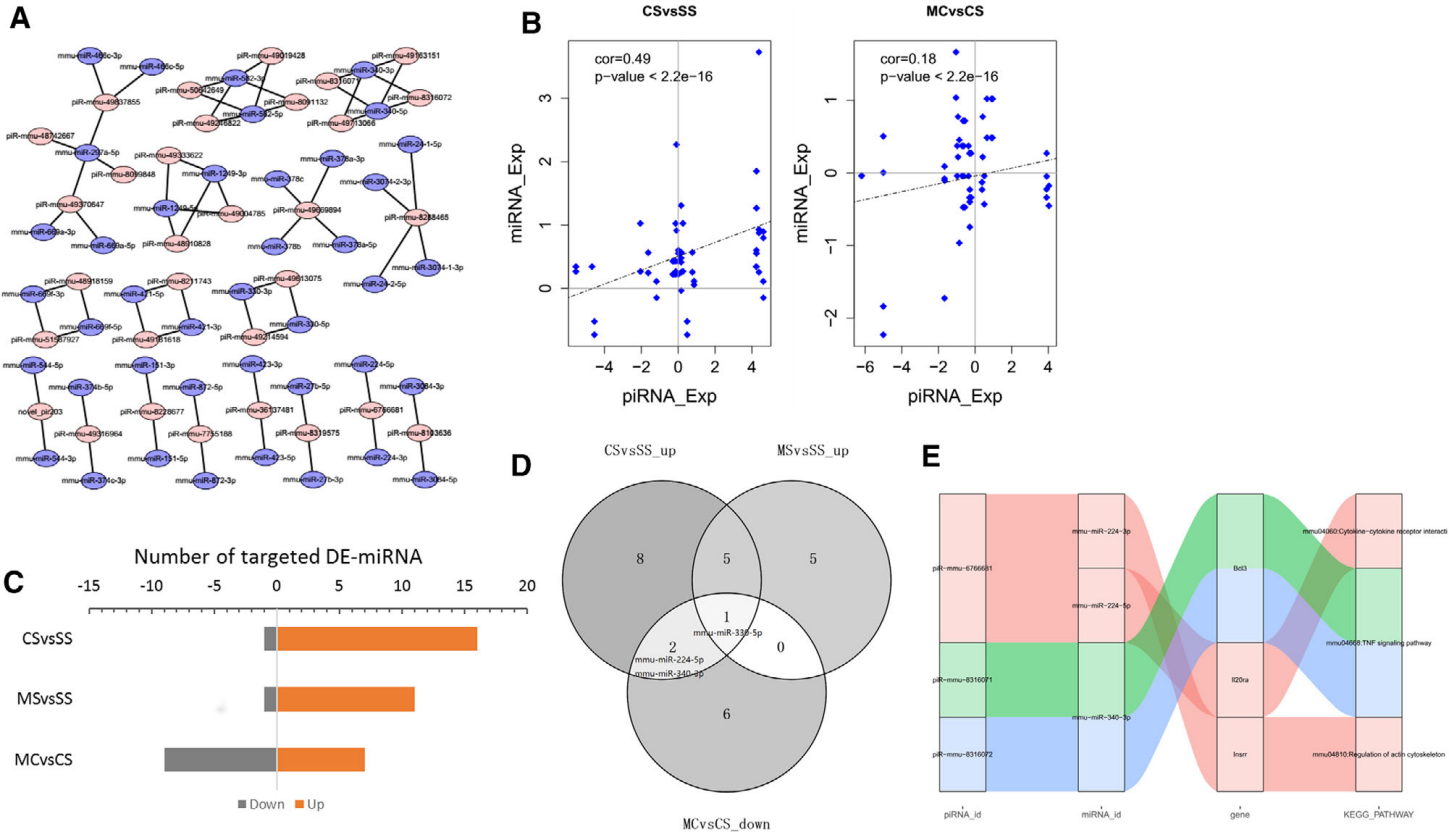
piRNA reaction to cocaine addiction and MET treatment response to cocaine addiction on mRNA genes through miRNAs. (a) Gene regulatory network of significantly regulated piRNAs and their potential target miRNAs. (b) Scatter plot of relationship of regulation between piRNAs and their potential target miRNAs of CSvsSS group and MCvsCS group. (c) Boxplot of the number of target DE‐miRNAs in each group. (d) Common target up‐regulated DE‐miRNAs in CSvsSS and MSvsSS groups and down‐regulated in MCvsCS group. (e) Sankey diagram of DE‐piRNA acted by MET treatment response to cocaine addiction with the target DE‐miRNAs, DEGs and pathways

According to base pair complementarity rules, we first performed network by significantly expressed piRNAs and their potential target miRNAs from our ncRNA sequencing data with cytoscape (v3.8.2) (Figure 5a). Mostly, unlike an mRNA gene can be targeted by multiple piRNAs, a miRNA can be targeted by only one or several piRNAs. This is probably because there are thousands and tons of piRNAs and miRNAs discovered in mice. Until now, the number of unique piRNA sequences in the mouse is over 68 million (Kim, 2019). Then we plotted the correlation of expression between piRNAs and their target miRNAs and showed there is a significantly positive correlation between piRNA and the potential target miRNA genes of CSvsSS group (cor = 0.49, *p*‐value < 2.20E‐16) and MCvsCS group (cor = 0.18, *p*‐value < 2.20E‐16) (Figure 5b).

To further investigate the mechanisms of piRNAs target miRNAs to regulate mRNAs of MET treatment for cocaine exposure, we first interrogated DE‐miRNAs whose expression changed in the same direction as the source piRNAs. There are 17 target DE‐miRNA in CSvsSS group, 12 target DE‐miRNA in MSvsSS and 16 target DE‐miRNA in MCvsCS(9 down‐regulated and 7 up‐regulated) (Figure 5c).

We then used up‐expressed of DE‐miRNs in CSvsSS, MSvsSS and down‐expressed of DE‐miRNAs in MCvsCS groups to do overlapping (Figure 5d). Venn diagram showed there are 2 common de‐miRNAs (mmu‐miR‐224‐5p, mmu‐miR‐340‐3p) between CSvsSS and MCvsCS; and 1 common de‐miRNA (mmu‐miR‐330‐5p) among three groups. Targeted mRNA gene in opposite expression changes of these three common DE‐miRNAs was identified and pathway analysis was done and plotted in Sankey diagram (Figure 5e). It showed there are three target genes located in three pathways respectively (Bcl3: mmu04668: TNF signaling pathway; Il20ra: mmu04060: Cytokine−cytokine receptor interaction; Insrr: mmu04810: Regulation of actin cytoskeleton). Interestingly, all three genes were reported to be associated with drug addiction (Freeman et al., 2010; Heller et al., 2015; Przybycien‐Szymanska et al., 2014). Especially, Bcl3 belongs to Bcl3‐NFKB2 complex which was reported to be involved in mediating complex behaviors including learning and memory, stress responses and drug reward (Nennig & Schank, 2017). This indicates that the MET can reverse cocaine effect via piRNAs which regulates gene expression through target miRNAs.

### 3.8 Validation of genes reversed by l‐methionine

**FIGURE 6:**
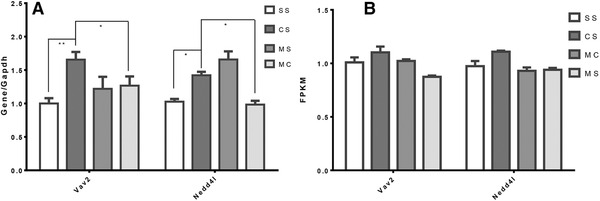
Expression of target genes regulated by piRNAs which was acted by cocaine and MET as determined by qPCR. (a) Normalized mRNA expression quantified by RT‐qPCR for genes whose expression is altered by cocaine treatment and reversed by MET: Vav2 and Nedd4l. Results represent mean ± SD of 3 determinations in each of the 2 types of experiments; ****p < 0.0001; ***p < 0.001; **p < 0.01; *p < 0.05. (b) Chart of fragments per kilobase of transcript per million mapped reads (FPKM) of Vav2 and Nedd4l from mRNA‐seq

Two gene of Vav2 (Vav Guanine Nucleotide Exchange Factor 2) and Nedd4l (Neural Precursor Cell Expressed, Developmentally Down‐Regulated 4‐Like) were identified targeted and regulated by DE‐piRNAs in the process of MET response of cocaine CPP. RT‐qPCR assay confirmed that expression of these two tested genes was significantly altered by cocaine and reversed by MET, which coincided with the results obtained from RNA‐sequencing (Figure 6).

## 4 DISCUSSION

Until recently, knowledge of the impact of abused drugs on gene and protein expression in the brain was still limited. As a class of important small ncRNA, piRNA gained a growing concern. More and more studies focused on their correlation with diseases. In this study, we assessed piRNA expression and tried to unclose the mechanism of piRNA in the process cocaine exposed and MET reversing cocaine‐CPP with ncRNA sequencing data. Of all sequencing data, 7.32% reads are piRNA. 86% of piRNAs located in repetitive elements: LINE1 (35%), LTR/ERVK (27%), and LTR/ERVL‐MaLR (24%). It was noted that piRNA usually matches repetitive elements, and gene expression regulated via repetitive elements is possible.

It was reported that the Piwi/piRNA complex regulates some protein‐coding genes in nervous system (Lee et al., 2011), whereas the mechanism of piRNA in the process of drug addiction is unknown. Our results showed that there was an inverse correlation between piRNA expression and its corresponding mRNA targets as was reported (Hashim et al., 2014). Pathway analysis showed that Morphine addiction, GABAergic synapse and Cholinergic synapse pathway were enriched of piRNA target mRNA genes. Meanwhile, we identified four genes—Cacna2d3, Epha6, Nedd4l, and Vav2—which were down‐regulated by piRNAs in the process of l‐methionine response for cocaine CPP. Thereinto, Vav2 was targeted by multiple piRNAs (piR‐mmu‐3777029, piR‐mmu‐22722094, piR‐mmu‐4568472, piR‐mmu‐49366621, piR‐mmu‐49443474 and piR‐mmu‐49341060) with the common target sequence: GTCTCTCCAGCCACCTT. It was noted that Vav2 deficiency has been reported to have diminished behavioral cocaine response (Zhu et al., 2015). While, piRNAs matched to repetitive elements and can suppress the activities of transposable elements at genomic, epigenetic levels and gene and protein regulation (L. Zuo et al., 2016). For example, PIWI silences gene by establishing a repressive chromatin state through increasing H3K9me3 and HP1. The mechanism on how piRNA regulate Vav2, such as via histone methylation, should be further investigated.

So far, there are few papers that discuss the regulation between miRNA and piRNA. In this paper, according to base pair complementarity rule, for the first time we found piRNA targeted miRNA with a possible positive correlation. At the same time, three down‐regulated DEGs were identified and regulated by piRNA through miRNA in the process of l‐methionine response of cocaine CPP: Bcl3, Il20ra and Insrr, which were reported to have an expression significantly regulated when drug acted on. Thus, l‐methionine treatment has an inverse response to cocaine administration via up‐regulating piRNAs and miRNA is possible.

In summary, our results revealed novel molecular mechanisms of MET that inhibited the rewarding actions of cocaine in brain reward circuitries, and provided a theoretic support for the development of anti‐addiction therapeutics based on MET.

## 5 CONCLUSIONS

Our results showed that piRNA negatively regulated target mRNA genes and positively regulated target miRNA genes. Genes located in substance dependence, signal transduction and nervous functions pathways were down‐regulated: Cacna2d3, Epha6, Nedd4l and Vav2. Thereinto, Vav2 was targeted by multiple piRNAs by sharing the common target sequence: GTCTCTCCAGCCACCTT. Taken together, these data may explain the roles of l‐methionine in counteracting the effects of cocaine CPP via piRNAs.

## CONFLICTS OF INTEREST

The authors have no conflicts of interest to declare.

## DATA ACCESSIBILITY

Non‐coding RNA‐seq gene expression data are deposited in NCBI GEO: Series GSE146631.

### PEER REVIEW

The peer review history for this article is available at https://publons.com/publon/10.1002/brb3.2272.

## AUTHORS CONTRIBUTION

Kunlin Zhang analyzed the data; Guanyu Ji analyzed the data and edited the article; Mei Zhao designed the study and interpreted the data; Yan Wang performed experiments, analyzed the data, and drafted the article.

## Supporting information

Supporting Information

Tables

## Data Availability

Data that support the findings of this study are available from the corresponding author upon reasonable request.
