# Supplementary material for: Candidate l‐methionine target piRNA regulatory networks analysis response to cocaine‐conditioned place preference in mice
Source: Brain Behav. 2021 Jul 1;11(8):e2272. doi: 10.1002/brb3.2272 (PMC8413732; doi:10.1002/brb3.2272)
Supplement: Supplementary file 1 — Supporting Information [file BRB3-11-e2272-s001.docx]

**Supplementary Table**1. Primer sequences used for rt-PCR.

| Primers used for QPCR | |
| --- | --- |
| Gene | Primer sequences for QPCR(5’-3’) |
| Vav2 | Fw: GTTTGACCCTTTCGACCTCTTT; Rv: ACACAGTCGTAGATGTCCTCA |
| CDT1 | Fw: GAGTCAAGGGGTTTTTGAGGTT; Rv: TGGGAAGCTGAGTCGTTGGA |

**Supplementary Table2**. List of differentially expressed piRNAs in CS, MS and MC groups (see Excel file table_s2).
